# Supplementary material for: From sequence to enzyme mechanism using multi-label machine learning
Source: BMC Bioinformatics. 2014 May 19;15:150. doi: 10.1186/1471-2105-15-150 (PMC4229970; doi:10.1186/1471-2105-15-150)
Supplement: Additional file 2 — Java code of ml2db. Additional file ml2db_code.tar.gz contains the Java source code to run the multi-label machine learning experiments and save the results to database. The code’s Javadoc is included. [file 1471-2105-15-150-S2.zip › additional file 2/ml2db/utils/doc/uk/ac/standrews/utils/main/TimeUtils.html]

TimeUtils


JavaScript is disabled on your browser.


- Overview
- Package
- Class
- Use
- Tree
- Deprecated
- Index
- Help

- Prev Class
- Next Class

- Frames
- No Frames

- All Classes

- Summary:
- Nested |
- Field |
- Constr |
- Method

- Detail:
- Field |
- Constr |
- Method


uk.ac.standrews.utils.main

## Class TimeUtils

- java.lang.Object
- - uk.ac.standrews.utils.main.TimeUtils

- ---

    

  ```
  public class TimeUtils
  extends java.lang.Object
  ```

  Utilities to manipulate time objects, create timestamps etc.

  Version:
  :   4 Jan 2008

  Author:
  :   Luna De Ferrari luna.deferrari-at-ed.ac.uk

- - ### Field Summary

    Fields

    | Modifier and Type | Field and Description |
    | --- | --- |
    | `static java.lang.String` | `DAY` |
    | `static java.lang.String` | `HOUR` |
    | `static java.lang.String` | `MILLISECONDS` |
    | `static java.lang.String` | `MINUTE` |
    | `static java.lang.String` | `MONTH` |
    | `static java.lang.String` | `SECOND` |
    | `static java.lang.String` | `YEAR` |
  - ### Constructor Summary

    Constructors

    Constructor and Description

    `TimeUtils()`
  - ### Method Summary

    Methods

    | Modifier and Type | Method and Description |
    | --- | --- |
    | `static java.lang.String` | `from1DigitTo2DigitsDate(java.lang.String date1digit)` Returns a day or month number in 2 digits format. |
    | `static java.lang.String` | `fromDMYYYtoYYYYMMDDdate(java.lang.String DMYYYYdate, java.lang.String inputDateSeparator, java.lang.String outputDateSeparator)` Converts from D/M/YYYY date (6/19/2006) to YYYY-MM-DD date (2006-06-19) |
    | `static java.lang.String` | `getDateForFileName()` getFileTimeStamp returns a date stamp that can be attached to file names |
    | `static java.lang.String` | `getDateHourStampForFileName()` Date-time stamp (up to hours) that can be attached to file names |
    | `static java.lang.String` | `getDateStamp()` Returns a date stamp in the format: 20070117 yyyymmdd |
    | `static java.util.HashMap<java.lang.String,java.lang.String>` | `getDateStrings()` getDateStrings() |
    | `static java.lang.String` | `getDateTimeMillisecondsStamp()` Returns a date-time-milliseconds stamp in the format: 20070117\_111847\_123 yyyymmdd\_hhmmss\_lll |
    | `static java.lang.String` | `getDateTimeNanoSecondsStamp()` Returns a date-time-milliseconds-nanoseconds stamp in the format: 20070117\_111847\_123\_3000 yyyymmdd\_hhmmss\_lll\_nnnn (the n digits are not the nanoseconds! just a progressive stamp) |
    | `static java.lang.String` | `getDateTimeStamp()` Returns a date-time stamp in the format: 20070117\_111847 yyyymmdd\_hhmmss |
    | `static java.lang.String` | `getElapsedTime(long startTime, long endTime)` Returns a string with the elapsed time in a hour:min:sec.msec format |
    | `static java.lang.String` | `getHourOnlyStamp()` Returns a time stamp in the format (for 11 am, 18 min, 47 sec): 11 hh |
    | `static java.lang.String` | `getHumanReadableTimeStamp()` Returns a human readable date-time stamp |
    | `static java.lang.String` | `getHumanReadableTimeStampMillisec()` |
    | `static java.lang.String` | `getMilliseconds()` Returns a milliseconds time stamp |
    | `static java.lang.String` | `getMillisecTimeStampForFileName()` Date-time stamp (up to milliseconds) that can be attached to file names |
    | `static java.lang.String` | `getNanoSecondsStamp()` Returns a nanoseconds stamp (it's a progressive stamp actually, not the actual nanoseconds in desktop-clock style!) |
    | `static java.lang.String` | `getNextNanoSecondsStamp(java.lang.String nanoTimeStamp)` Returns a nanoseconds stamp + 1 (it's a progressive stamp actually, not the actual nanoseconds in desktop-clock style!) |
    | `static java.sql.Timestamp` | `getSqlTimestamp()` Gets a sql.Timestamp object of the current time (precise to the second: nanoseconds are set to zero) |
    | `static java.lang.String` | `getSqlTimestampString()` |
    | `static java.lang.String` | `getTimeOnlyStamp()` Returns a time stamp in the format (for 11 am, 18 min, 47 sec): 111847 hhmmss |
    | `static java.lang.String` | `getTimePassed(java.util.Calendar startTime)` |
    | `static java.lang.String` | `getTimeStampForFileName()` Date-time stamp (up to seconds) that can be attached to file names |
    | `static void` | `printTimestampedComment(java.lang.String comment)` Writes to System.out a timestamp + tab + the comment. |
    | `static void` | `printTimestampedError(java.lang.String comment)` |
    | `static java.lang.String` | `timestampedComment(java.lang.String comment)` |

    - ### Methods inherited from class java.lang.Object

      `equals, getClass, hashCode, notify, notifyAll, toString, wait, wait, wait`

- - ### Field Detail


    - #### DAY

      ```
      public static final java.lang.String DAY
      ```

      See Also:
      :   Constant Field Values


    - #### HOUR

      ```
      public static final java.lang.String HOUR
      ```

      See Also:
      :   Constant Field Values


    - #### MILLISECONDS

      ```
      public static final java.lang.String MILLISECONDS
      ```

      See Also:
      :   Constant Field Values


    - #### MINUTE

      ```
      public static final java.lang.String MINUTE
      ```

      See Also:
      :   Constant Field Values


    - #### MONTH

      ```
      public static final java.lang.String MONTH
      ```

      See Also:
      :   Constant Field Values


    - #### SECOND

      ```
      public static final java.lang.String SECOND
      ```

      See Also:
      :   Constant Field Values


    - #### YEAR

      ```
      public static final java.lang.String YEAR
      ```

      See Also:
      :   Constant Field Values
  - ### Constructor Detail


    - #### TimeUtils

      ```
      public TimeUtils()
      ```
  - ### Method Detail


    - #### from1DigitTo2DigitsDate

      ```
      public static java.lang.String from1DigitTo2DigitsDate(java.lang.String date1digit)
      ```

      Returns a day or month number in 2 digits format. Example: month 5
      becomes month 05.

      Parameters:
      :   `date1digit` -

      Returns:
      :   a day or month number in 2 digits format


    - #### fromDMYYYtoYYYYMMDDdate

      ```
      public static java.lang.String fromDMYYYtoYYYYMMDDdate(java.lang.String DMYYYYdate,
                                             java.lang.String inputDateSeparator,
                                             java.lang.String outputDateSeparator)
      ```

      Converts from D/M/YYYY date (6/19/2006) to YYYY-MM-DD date (2006-06-19)

      Parameters:
      :   `DMYYYYdate` - a D M YYYY date (example: 6/19/2006)
      :   `inputDateSeparator` - (example: / )
      :   `outputDateSeparator` - (example: - )

      Returns:
      :   a YYYY MM DD date (2006-06-19)


    - #### getDateForFileName

      ```
      public static java.lang.String getDateForFileName()
      ```

      getFileTimeStamp returns a date stamp that can be attached to file names

      Returns:
      :   a string with date in the format: \_20070117 \_yyyymmdd)


    - #### getDateHourStampForFileName

      ```
      public static java.lang.String getDateHourStampForFileName()
      ```

      Date-time stamp (up to hours) that can be attached to file names

      Returns:
      :   a string with date and hour in the format: \_20070117\_11
          \_yyyymmdd\_hh)


    - #### getDateStamp

      ```
      public static java.lang.String getDateStamp()
      ```

      Returns a date stamp in the format: 20070117 yyyymmdd

      Returns:
      :   a string with date in the format: 20070117 yyyymmdd)


    - #### getDateStrings

      ```
      public static java.util.HashMap<java.lang.String,java.lang.String> getDateStrings()
      ```

      getDateStrings()

      Returns:
      :   a hashmap containing the date-time elements as strings


    - #### getDateTimeMillisecondsStamp

      ```
      public static java.lang.String getDateTimeMillisecondsStamp()
      ```

      Returns a date-time-milliseconds stamp in the format: 20070117\_111847\_123
      yyyymmdd\_hhmmss\_lll

      Returns:
      :   a date-time-milliseconds stamp in the format: 20070117\_111847\_123
          yyyymmdd\_hhmmss\_lll


    - #### getDateTimeNanoSecondsStamp

      ```
      public static java.lang.String getDateTimeNanoSecondsStamp()
      ```

      Returns a date-time-milliseconds-nanoseconds stamp in the format:
      20070117\_111847\_123\_3000 yyyymmdd\_hhmmss\_lll\_nnnn (the n digits are not
      the nanoseconds! just a progressive stamp)

      Returns:
      :   a date-time-milliseconds stamp in the format: 20070117\_111847\_123
          yyyymmdd\_hhmmss\_lll\_n


    - #### getDateTimeStamp

      ```
      public static java.lang.String getDateTimeStamp()
      ```

      Returns a date-time stamp in the format: 20070117\_111847 yyyymmdd\_hhmmss

      Returns:
      :   a string with date in the format: 20070117\_111847 yyyymmdd\_hhmmss


    - #### getElapsedTime

      ```
      public static java.lang.String getElapsedTime(long startTime,
                                    long endTime)
      ```

      Returns a string with the elapsed time in a hour:min:sec.msec format

      Parameters:
      :   `startTime` - a time in milliseconds (System.currentTimeMillis())
      :   `endTime` - a time in milliseconds (System.currentTimeMillis())

      Returns:


    - #### getHourOnlyStamp

      ```
      public static java.lang.String getHourOnlyStamp()
      ```

      Returns a time stamp in the format (for 11 am, 18 min, 47 sec): 11 hh

      Returns:
      :   a string with time in the format (for 11 am, 18 min, 47 sec): 11
          hh


    - #### getHumanReadableTimeStamp

      ```
      public static java.lang.String getHumanReadableTimeStamp()
      ```

      Returns a human readable date-time stamp

      Returns:
      :   a string with date and time in the format: YYYY-MM-DD hh:mm:ss


    - #### getHumanReadableTimeStampMillisec

      ```
      public static java.lang.String getHumanReadableTimeStampMillisec()
      ```


    - #### getMilliseconds

      ```
      public static java.lang.String getMilliseconds()
      ```

      Returns a milliseconds time stamp

      Returns:
      :   a milliseconds time stamp


    - #### getMillisecTimeStampForFileName

      ```
      public static java.lang.String getMillisecTimeStampForFileName()
      ```

      Date-time stamp (up to milliseconds) that can be attached to file names

      Returns:
      :   a string with date and time in the format: \_20070117\_111847
          \_yyyymmdd\_hhmmss)


    - #### getNanoSecondsStamp

      ```
      public static java.lang.String getNanoSecondsStamp()
      ```

      Returns a nanoseconds stamp (it's a progressive stamp actually, not the
      actual nanoseconds in desktop-clock style!)

      Returns:
      :   a nanoseconds stamp


    - #### getNextNanoSecondsStamp

      ```
      public static java.lang.String getNextNanoSecondsStamp(java.lang.String nanoTimeStamp)
      ```

      Returns a nanoseconds stamp + 1 (it's a progressive stamp actually, not
      the actual nanoseconds in desktop-clock style!)

      Returns:
      :   a nanoseconds stamp


    - #### getSqlTimestamp

      ```
      public static java.sql.Timestamp getSqlTimestamp()
      ```

      Gets a sql.Timestamp object of the current time (precise to the second:
      nanoseconds are set to zero)

      Returns:
      :   a sql.Timestamp object of the current time


    - #### getSqlTimestampString

      ```
      public static java.lang.String getSqlTimestampString()
      ```


    - #### getTimeOnlyStamp

      ```
      public static java.lang.String getTimeOnlyStamp()
      ```

      Returns a time stamp in the format (for 11 am, 18 min, 47 sec): 111847
      hhmmss

      Returns:
      :   a string with time in the format (for 11 am, 18 min, 47 sec):
          111847 hhmmss


    - #### getTimePassed

      ```
      public static java.lang.String getTimePassed(java.util.Calendar startTime)
      ```


    - #### getTimeStampForFileName

      ```
      public static java.lang.String getTimeStampForFileName()
      ```

      Date-time stamp (up to seconds) that can be attached to file names

      Returns:
      :   a string with date and time in the format: \_20070117\_111847
          \_yyyymmdd\_hhmmss)


    - #### printTimestampedComment

      ```
      public static void printTimestampedComment(java.lang.String comment)
      ```

      Writes to System.out a timestamp + tab + the comment.

      Parameters:
      :   `comment` -


    - #### printTimestampedError

      ```
      public static void printTimestampedError(java.lang.String comment)
      ```


    - #### timestampedComment

      ```
      public static java.lang.String timestampedComment(java.lang.String comment)
      ```


- Overview
- Package
- Class
- Use
- Tree
- Deprecated
- Index
- Help

- Prev Class
- Next Class

- Frames
- No Frames

- All Classes

- Summary:
- Nested |
- Field |
- Constr |
- Method

- Detail:
- Field |
- Constr |
- Method
